# Supplementary material for: Real world perspective of coronary chronic total occlusion in third world countries: A tertiary care centre study from northern India
Source: Indian Heart J. 2021 Mar 17;73(2):156–60. doi: 10.1016/j.ihj.2021.03.001 (PMC8065346; doi:10.1016/j.ihj.2021.03.001)
Supplement: Multimedia component 1 [file mmc1.docx]

**REAL WORLD PERSPECTIVE OF CORONARY CHRONIC TOTAL OCCLUSION IN THIRD WORLD COUNTRIES: A TERTIARY CARE CENTRE STUDY FROM NORTHERN INDIA**

**Abstract**

**Objectives:**  The aim of this study is to determine the prevalence, clinical characteristics, angiographic profile and predictors of outcome for percutaneous coronary interventions (PCI) of coronary chronic total occlusions (CTO) in a tertiary referral centre of north India.

**Background:** There is no data on the prevalence and very few reports on clinical characteristics, angiographic profile and outcome of PCI in CTO from India.

**Methods:** Retrospective analysis was done for the data of 12020 patients undergoing coronary angiography (CAG) between January 2018 to January 2019 at our centre. Detailed baseline clinical, angiographic and revascularization data was collected. Outcome of CTO PCI was also noted. All baseline parameters were analysed for predicting the outcome of CTO PCI.

**Results:** CTO was identified in 16.3% (1968) patients undergoing CAG and in 24.4% of patients with hemodynamically significant CAD. CTO was predominantly found in LAD (48 %) followed by RCA (42.9%) and LCx (25.3%) arterial distribution. Mean JCTO score was 1.93 ± 0.7. PCI as a management strategy was adopted in 456 of 1968 patients (23.1%) and was successful in 340 of 456 (74.6%) of patients. Almost all CTO PCI were attempted by an antegrade approach only. Increasing age, male sex, CTO in LCx arterial distribution and higher J CTO score were associated with poorer outcome in CTO PCI.

**Conclusions:** CTO’s are commonly encountered during CAG procedures. In patients undergoing CTO PCI, a fair success rate can be achieved in a high volume experienced centre.

**Key Words**: Coronary Artery Disease (CAD), Chronic total occlusion (CTO), J CTO Score, Percutaneous Coronary Intervention (PCI).

**REAL WORLD PERSPECTIVE OF CORONARY CHRONIC TOTAL OCCLUSION IN THIRD WORLD COUNTRIES: A TERTIARY CARE CENTRE STUDY FROM NORTHERN INDIA**

**Introduction**

Chronic total occlusion (CTO) is defined as 100% obstruction to antegrade flow in the coronary arteries for at-least three months duration.^1^ Reports in the past have shown variable prevalence of CTO in diagnostic angiography.^2,3,4,5^ The true prevalence of CTO is not known as it depends on the reason for which CAG is being performed. Performing a percutaneous coronary intervention (PCI) of CTO has always been challenging. It requires a high amount of expertise, acquired skills and dedicated training, along with ever enlarging armamentarium of hardware. The success rate of CTO PCI is also variable.^6,7,8,9,10^ The New York state PCI registry and U.S National Cardiovascular Data Registry (NCDR) shows a success rate of CTO PCI to be 61% and 59% respectively.^6,7^ But, with increasing expertise (use of contemporary equipment’s and techniques especially the retrograde and hybrid algorithm) higher success rates (86%-90%) have been documented.^8,9,10^ Thus, there has been a significant gap in the results achieved for CTO PCI even in developed nations.

To the best of our knowledge there has been no data on prevalence of CTO from India and also there are only few reports on CTO PCI success rate and its predictors.^11,12,13,14^ Thus, it becomes especially important for us to have this knowledge in resource limited countries like India where there is a limited coverage of public health insurance and very few people can afford these procedures due to financial constraints and lack of adequate technical expertise. In the current study we planned to determine the true prevalence of CTO in all comer population undergoing CAG at a tertiary referral centre of north India and also to determine the predictors of success in CTO PCI.

1. **Material and Methods**
   1. **Aim**

The primary aim of the study is to determine the prevalence, clinical characteristics and angiographic profiles of CTO in all comer population undergoing CAG. Secondarily we also would like to determine outcomes of CTO-PCI and predictors of its success.

- 1. **Study design**

The study is a retrospective analysis of all CAG’s performed at our centre since January 2018 till February 2019.

- 1. **Definition of CTO and parameters noted**

Any patient with chronic stable angina with symptom duration more than three months with 100% occlusion in any of the three major arterial distribution of coronary circulation was defined as CTO. In cases of ACS, if there was a 100% occluded artery in the arterial distribution different from the artery causing ACS (as corroborated with electrocardiogram) it was labelled as CTO. In all CAG’s following parameters were noted: distribution of atherosclerosis, dominance of coronary circulation (left, right or co-dominant), SYNTAX score and if a CTO is found a detailed analysis of CTO artery was performed and J-CTO score was calculated.

- 1. **J-CTO score calculation^15^**

For every confirmed CTO, J-CTO score was determined. It was calculated using a 5 point scoring system considering following parameters: 1.Blunt stump; 2.Calcification; 3.Within lesion bending more than 45 degrees; 4.Occlusion length more than equal to 20 mm; 5.Prior failed attempt to revascularize the CTO. Each parameter was given a score of 1.

**Difficulty level for intervention^15^:** Score zero was considered easy, 1 as intermediate, 2 as difficult and any score more than or equal to 3 was considered very difficult for successful PCI of CTO.

**Decision to revascularize:** As it is a retrospective analysis we did not have any control on the primary operator’s decision to consider medical management, perform PCI or send for CABG. Myocardial viability status whether performed prior to PCI was determined on retrospective review of hospital records.

**Procedural techniques for CTO intervention:** At the outset we accept that none of our operators have an experience of performing routine retrograde PCI for CTO and thus all except 3 cases were performed by antegrade technique of CTO interventions only. The predominant technique used for the procedure was antegrade guidewire escalation with or without antegrade parallel wire technique. Complex and skilled techniques like antegrade dissection and re-entry (ADR), subintimal tracking and re-entry (STAR), controlled antegrade and retrograde subintimal tracking (CART) and various retrograde techniques were used only in 3 patients.

**Parameters analysed:** Demographic and clinical parameters were noted for all patients. Both successful and unsuccessful CTO-PCI were studied and analysed with respect to their SYNTAX, J-CTO scores and other angiographic characteristics. The other details such as number and type of hardware used, fluoroscopy time and radiation exposure were not analysed in this study due to difficulties in procuring these details. Success of the procedure was defined as the ability to successfully cross the CTO segment with guidewire and restoration of TIMI flow grade 2 or 3 in the distal vessel after PCI. Complications such as coronary artery perforation, cardiac tamponade, peri procedural myocardial infarction, major bleeding and death were also recorded. The follow-up details were only available till there discharge from the hospital in the index hospitalization. No further follow-up details were obtained for analysis.

**Statistical analysis**

Continuous variables were expressed as mean ± standard deviation and categorical variables as percentages or frequencies. The variables were tested for normality of distribution of values. The differences were compared using chi square test, student t test, and Mann- Whitney test wherever appropriate. Comparison was performed among the CTO procedures and compared the characteristics of successful versus non successful CTO PCI. Predictors for successful CTO PCI were analysed initially using univariate logistic regression analyses and those that were significant (p < 0.1) were subjected to multivariate regression analysis to detect the independent predictors. All tests were 2 sided and p value <0.05 was considered statistically significant. Statistical analysis was performed using SPSS software (Version 26, Chicago, IL, USA).

**Results**

A total of 12020 consecutive CAG’s and 4600 PCI performed during the above mentioned period were analysed. Baseline parameters are provided in table 1. The study population shows a male preponderance, with 8724 (72.5%) of them being male. The risk factors like Diabetes Mellitus (DM), hypertension (HT), smoking, and family history were seen in 44.6%, 51.6%, 24%, and 13.9% patients respectively. Among angiographic parameters, normal coronaries or insignificant (< 50% obstruction) coronary artery disease CAD was seen in 32.8% of patients. Single, double and triple vessel involvement was seen in 32.1 %, 20.4 %, 14.6 % of overall angiograms respectively and 47.8 %, 30.4 %, 21.7 % among patients with significant CAD (any artery showing luminal stenosis >50% diametric stenosis). Left main coronary involvement was seen in 4.2 % of overall angiograms and 6.2 % in subjects with significant CAD. Right dominant circulation accounted for 83.5%, left and co dominant circulation were seen in 10.7%, 5.7% respectively. Regarding diseased vessels, left anterior descending (LAD) artery, left circumflex (LCx) and right coronary artery (RCA) were significantly diseased (>50% stenosis) in 71.6%, 48.4%, 50.8% of patients. SYNTAX score was calculated in all patients with significant CAD. Scores of less than 22 seen in 78.9% of patients, scores 22 to 32 seen in 12.9 % and score more than 32 seen in 8.2 % of patients.

CTO were observed in 1968/12020 (16.3%) of all patients undergoing CAG~~,~~ and 24.4% among patients with significant CAD. Amongst all CTO’s, LAD CTO accounts for 48.1% cases, and RCA accounts for 42.9%, and LCx accounts for 25.2% cases.

In all patients with CTO, PCI was attempted in 456 (23.1%) patients and was successful in 340 (74.6%) patients. However, CTO PCI accounted for 10 % of all PCI performed in the above mentioned period. Almost all the cases (99.3%) were performed by antegrade wire escalation or parallel wire technique and only 3 (0.65%) patients underwent retrograde technique. Radial artery access (for performing PCI or for contralateral artery engagement) was obtained in 164 (36%) of cases only.

Out of 456 CTO PCI attempted, the operators were able to achieve a success in 340 (74.5%) of cases. Comparison between the successful and unsuccessful CTO-PCI is shown in **table 2**. Patients with successful CTO-PCI were younger, had low J-CTO score and had predominantly single vessel involvement. Patients with unsuccessful CTO-PCI had more multivessel involvement and CTO in LCx artery distribution. Though the mean SYNTAX score was similar between the groups, patients with score more than 22 were higher in the unsuccessful group. None of the other baseline demographics or risk factors were different between the groups.

Multivariate logistic regression analysis was performed to determine the independent predictors of CTO-PCI. It showed higher age, higher J CTO score, male sex and presence of CTO in LCx were negatively associated with success of CTO-PCI.

**Periprocedural complications**:

There were 4 (0.9%) procedure related deaths during the index hospitalization in the entire cohort. Two deaths occurred during the procedure and two deaths during the course of hospital stay. Mild pericardial effusion not amounting to tamponade was seen in 12 (2.6%) of cases and cardiac tamponade requiring pericardiocentesis was seen in 3 (0.6%) patients. Local site complications like femoral hematoma was seen in 10 (2.2%) patients, pseudoaneurysm in 4 (0.9%) of them which was managed conservatively during the hospital stay. Coronary artery perforations were noted in 6 (1.3%) patients. No retroperitoneal hematoma or major bleeding were seen in the entire cohort.

**Discussion**

To the best of our knowledge this is the first study to demonstrate prevalence of CTO and its outcomes in an all-comer population of North India. The study is done in a very high volume tertiary referral centre of Northern India. Amongst all-comer population of 12020 CAG’s, we found that the prevalence of CTO to be 16.3%. Reported prevalence of CTO in prior studies is highly variable.^2,3,4,5^ Highest prevalence of 52% has been reported from University of Washington in patients with significant (>70% luminal stenosis) CAD.^2^ However, our data highly resembles to the Canadian multicentre CTO registry data.^3^ They found the true prevalence of CTO in all comer population to be 14.7% and 18.4% in patients with established CAD. This study is a prospective registry data from 3 Canadian institutes. A French multicentre registry of unselected population shows prevalence of CTO to be 8.1%. This significant discrepancy in the data may be attributed to indication for performing an angiography and also on the prevalence of CAD in the population being studied. As such Indian population has an early onset of CAD and prevalence of diffuse CAD. Higher incidence in our study compared to the contemporary registry data may also be because of inclusion of all patients, even with acute coronary syndrome (ACS), which have been excluded in other studies. It has been found that about 10%-15% of patients with STEMI are found to have a CTO, apart from the culprit vessel for MI.^16,17^ Another reason for higher prevalence may be a lack of overall health infrastructure in India leading to a very low percentage of primary PCI for STEMI. Thus these vessels may over a period of time go on to become CTO and diagnosed later on.

Management strategies for CTO PCI are also variable. PCI as a treatment strategy for CTO revascularization was performed in 49% of patients in a French registry data and 11% in American study.^2,18^ In our study PCI by revascularization was attempted in 23.1% of all CTO’s detected and overall CTO PCI accounted for 10% of total PCI during the study period. US NCDR data shows that CTO PCI accounts for just 3.2 to 4.8% of all PCI and Canadian registry shoes 30% of all CTO patients underwent PCI.^3,7^ Our study shows an overall similar percentage compared to Canadian data but higher than the American data. The important postulated reason for this at our centre is because of a longer waiting list for patients to undergo coronary artery bypass surgery (CABG) and thus patients opting for PCI as an alternative therapy because of recurrent angina.

We found an overall success rate of CTO PCI to be 74.5%. This appears to be highly promising in view of an average JCTO score of all attempted cases to be 1.78±0.70 which is between intermediate and difficult levels in terms of chances of success.^15^ This is further substantiated by the fact that almost all cases were performed by antegrade techniques only. Result of CTO PCI is highly variable in the international literature.^6,7,8,9,10^ The New York state PCI registry and U.S NCDR data shows a success rate of CTO PCI to be 61% and 59% respectively.^6,7^ A recent data from multicentre registry demonstrates the success rate of antegrade only technique to be 64% and 47% in patients with JCTO score of 1 and 2 respectively.^19^ But, with increasing expertise with use of contemporary equipment and techniques especially the retrograde and hybrid algorithm can achieve a higher success rates of 86%-90%.^8,9,10^ The US NCDR data says that the success of CTO PCI depends upon the operator's experience. For operators performing <5, 5 to 10, and >10 CTO PCI per year the success rate was found to be 53%, 62%, and 75% respectively. Comparing this to our centre data, overall we performed 456 CTO PCI in a year with around 10 operators performing CTO PCI at the centre. Thus, we fulfil the criteria of being a very high volume centre for CTO interventions, with a fair success rate compared to developed nations. We also compared our study results with the existing data from India.^11,13,13,14^ Overall the results from prior Indian studies show a CTO PCI success rate to be 68 to 87%. The largest data of 632 patients is by Goel et al collected over a period of 9 years.^13^ The success rate of CTO PCI was 74.7% and the clinical and angiographic profile of patients were very similar to our study population. The average JCTO score was 1.5 compared to 1.78 in our study. Another data from high volume centre of south India showed a CTO PCI success rate of 87% in selected population with an average JCTO score of 1.78 ± 0.12. This study showed around 10% of cases were performed by retrograde technique which was utilized in <1% of our cases thus may affect the overall success rate of our CTO PCI.^11^ Thus, we still have a long way to go in achieving a higher success rate in CTO PCI compared to very highly skilled operating centres where the success rate of almost 90% is achieved routinely.^20^

Our study also showed advanced age, male sex, increasing JCTO score, CTO in LCx distribution to be independently associated with poor procedural success of CTO PCI. Only one study from India have studied the predictors of procedural outcome in CTO PCI and found high JCTO score and history of unstable angina as independent predictors of unsuccessful CTO PCI.^11^ However, various clinical and angiographic scores have been developed in the past to predict the success of CTO PCI.^21^ They have shown prior CABG, prior MI, severe calcification, long length of CTO, non-LAD CTO and blunt stump to be predictors of failure of CTO PCI. Another novel score developed for antegrade only technique have taken only lesion characteristics such as bridging collaterals, absence of stump, calcification, acute bend, presence of side branch, and lack of retrograde filling as predictors of poor outcome in CTO PCI.^22^ Thus, overall the JCTO score takes into account lesion characteristics and added on to it the clinical features can predict the outcome of CTO PCI. Male sex and advanced age are new clinical predictors found in our study to be negatively associated with CTO PCI success. The strength of such an association can be evaluated in future prospective clinical study.

**Limitations**

As an observational study it can just find the association between the parameters and procedural outcome and cannot determine the causality with confidence. As it is a single centre experience with a very high volume of overall PCI and CTO PCI, a bias may arise because of patients being referred to us and may overestimate the prevalence. Also as multiple interventionists at various levels of expertise were involved in management, their treatment decisions and operative characteristics would have influenced the studied parameters and outcomes and may not be a true representation of CTO PCI outcomes in current era. Another limitation of the study is lack of demonstration of myocardial viability prior to decision making in the treatment of CTO. In view of the retrospective nature of study design, we did not evaluate the improvement of clinical characteristics and MACE (Major Adverse Cardiovascular Events) and TLR (Target Lesion Revascularization) of study population which we consider a major limitation of this study. Our study population did not had usage of invasive imaging like IVUS (Intravascular Ultrasound) or OCT (Optical coherence tomography). Nevertheless, the study determines the prevalence of CTO in India for the first time and also determines its clinical and angiographic characteristics.

**Conclusion**

The present study provides vital information of CTO prevalence in contemporary practice of catheterization laboratory in India. It also describes in detail about its clinical, angiographic features and predictors of outcome of CTO PCI. It has found that in patients of CAD nearly one quarter will have an artery with CTO and fair success in PCI of CTO can be attained by antegrade technique in an experienced setting.

**Acknowledgement**

None

**Disclosure**

Nothing to disclose

**Funding Sources**

None.

**References**

1. Tajti P, Burke MN, Karmpaliotis D, Alaswad K, Werner GS, Azzalini L, Carlino M, Patel M, Mashayekhi K, Egred M, Krestyaninov O, Khelimskii D, Nicholson WJ, Ungi I, Galassi AR, Banerjee S, Brilakis ES. Update in the percutaneous management of coronary chronic total occlusions. *JACC Cardiovasc Interv*. 2018;11:615–625. doi: 10.1016/j.jcin.2017.10.052
2. Christofferson RD, Lehmann KG, Martin GV, Every N, Caldwell JH, Kapadia SR. Effect of chronic total coronary occlusion on treatment strategy. *Am J Cardiol* 2005;95:1088–1091.
3. Fefer P, Knudtson ML, Cheema AN, Galbraith PD, Osherov AB, Yalonetsky S, Gannot S, Samuel M, Weisbrod M, Bierstone D, Sparkes JD, Wright GA, Strauss BH. Current perspectives on coronary chronic total occlusions: the Canadian Multicenter Chronic Total Occlusions Registry. *J Am Coll Cardiol* 2012;59:991–997.
4. Jeroudi OM, Alomar ME, Michael TT, Sabbagh AE, Patel VG, Mogabgab O, Fuh E, Sherbet D, Lo N, Roesle M, Rangan BV, Abdul- lah SM, Hastings JL, Grodin J, Banerjee S, Brilakis ES. Prevalence and management of coronary chronic total occlusions in a tertiary veterans affairs hospital. *Catheter Cardiovasc Interv* 2014;84:637–643.
5. Ramunddal T, Hoebers LP, Henriques JPS, Dworeck C, Angeras O, Odenstedt J, Ioanes D, Olivecrona G, Harnek J, Jensen U, Aasa M, Albertsson P, Wedel H, Omerovic E. Prognostic impact of chronic total occlusions: a report from SCAAR (Swedish Coronary Angiography and Angioplasty Registry). *JACC Cardiovasc Interv* 2016;9:1535–1544.
6. Hannan EL, Zhong Y, Jacobs AK, et al. Patients with chronic total occlusions undergoing percu- taneous coronary interventions: characteristics, success, and outcomes. *Circ Cardiovasc Interv* 2016;9:e003586.
7. Brilakis ES, Banerjee S, Karmpaliotis D, et al. Procedural outcomes of chronic total occlusion percutaneous coronary intervention: a report from the NCDR (National Cardiovascular Data Registry). *J Am Coll Cardiol Interv* 2015;8:245–53.
8. Christopoulos G, Karmpaliotis D, Alaswad K, et al. Application and outcomes of a hybrid approach to chronic total occlusion percutaneous coronary intervention in a contemporary multi- center US registry. *Int J Cardiol* 2015;198:222–8.
9. Habara M, Tsuchikane E, Muramatsu T, et al. Comparison of percutaneous coronary interven- tion for chronic total occlusion outcome according to operator experience from the Japanese retro- grade summit registry. *Catheter Cardiovasc Interv* 2016;87:1027–35.
10. Maeremans J, Walsh S, Knaapen P, et al. The hybrid algorithm for treating chronic total occlu- sions in Europe: the RECHARGE registry. *J Am Coll Cardiol* 2016;68:1958–70.
11. Pillai AA, Ramasamy S, Jagadheesan KS, Satheesh S, Selvaraj RJ, Jayaraman B. Procedural and follow-up clinical outcomes after chronic total occlusion revascularization: Data from an Indian public hospital. *Indian heart journal* 2019 Jan 1;71(1):65-73.
12. Gopakumar KS, Iype M, Viswanathan S, Koshy AG, Gupta PN, Sivaprasad K, Radhakrishnan VV. Angiographic predictors of success in antegrade approach of Chronic Total Occlusion interventions in a South Indian population in the contemporary era. *Indian heart journal* 2018 Jan 1;70(1):15-9.
13. Goel PK, Khanna R, Pandey CM, Ashfaq F. Long‐term outcomes post chronic total occlusion intervention—implications of completeness of revascularization. *Journal of interventional cardiology* 2018 Jun;31(3):293-301.
14. Mehta AB, Mehta N, Chhabria R, Mandurke V, Tawade N, Jain N, Desai A, Handa SR. Predictors of success in percutaneous Coronary intervention for chronic total occlusion. *Indian Heart Journal* 2018 Dec; 70(Suppl 3): S269–S274.
15. Morino Y, Abe M, Morimoto T, Kimura T, Hayashi Y, Muramatsu T, et al. Predicting successful guide- wire crossing through chronic total occlusion of native coronary lesions within 30 minutes: the J-CTO (Multicenter CTO Registry in Japan) score as a difficulty grading and time assessment tool. *JACC Cardiovasc Interv*. 2011; 4:213–221.
16. Claessen BE, Dangas GD, Weisz G, Witzenbichler B, Guagliumi G, M€ockel M, Brener SJ, Xu K, Henriques JPS, Mehran R, Stone GW. Prognostic impact of a chronic total occlusion in a non-infarct-related artery in patients with ST-segment elevation myocardial infarction: 3-year results from the HORIZONS-AMI trial. *Eur Heart J* 2012;33:768–775.
17. Claessen BEPM, van der Schaaf RJ, Verouden NJ, Stegenga NK, Eng- strom AE, Sjauw KD, Kikkert WJ, Vis MM, Baan J, Koch KT, de Winter RJ, Tijssen JGP, Piek JJ, Henriques JPS. Evaluation of the effect of a concurrent chronic total occlusion on long-term mortality and left ventricular function in patients after primary percutaneous cor- onary intervention. *JACC Cardiovasc Interv* 2009;2:1128–1134.
18. Boukantar M, Loyeau A, Gallet R, Bataille S, Hakim Benamer H, Caussin C, Garot P, Livarek B, Varenne O, Spaulding C, Karrillon G, Emmanuel Teiger E. Angiography and Percutaneous Coronary Intervention for Chronic Total Coronary Occlusion in Daily Practice (from a Large French Registry [CARDIO-ARSIF]). *Am J Cardiol* 2019;124:688−695.
19. Christopoulos G, Wyman RM, Alaswad K, Karmpaliotis D, Lombardi W, Grantham JA, Yeh RW et al. Clinical Utility of the J-CTO Score in Coronary Chronic Total Occlusion Interventions: Results from a Multicenter Registry *Circ Cardiovasc Interv.* 2015 Jul; 8(7), e002171,2015
20. Suzuki Y, Tsuchikane E, Katoh O, Muramatsu T, Muto M, Kishi K, et al. Outcomes of Percutaneous Coronary Interventions for Chronic Total Occlusion Performed by Highly Experienced Japanese Spe- cialists: The First Report From the Japanese CTO-PCI Expert Registry. *JACC Cardiovasc Interv.* 2017; 10:2144–2154.
21. Alessandrino G, Chevalier B, Lefèvre T, Sanguineti F, Garot P, Unterseeh T, Hovasse T, Morice MC, Louvard Y. A Clinical and Angiographic Scoring System to Predict the Probability of Successful First-Attempt Percutaneous Coronary Intervention in Patients With Total Chronic Coronary Occlusion *JACC Cardiovasc Interv* 2015 Oct;8(12):1540-8.
22. Namazi MH, Serati AR, Vakili H, Safi M, Parsa SAP, Saadat H, Taherkhani M, Emami S, Pedari S, Vatanparast M, Movahed MR. A Novel Risk Score in Predicting Failure or Success for Antegrade Approach to Percutaneous Coronary Intervention of Chronic Total Occlusion: Antegrade CTO Score. *Int J Angiol.* 2017 Jun; 26(2): 89–94.
